# Association of serum osteoprotegerin with vascular calcification in patients with type 2 diabetes

**DOI:** 10.1186/1475-2840-12-11

**Published:** 2013-01-09

**Authors:** Atsushi Aoki, Miho Murata, Tomoko Asano, Aki Ikoma, Masami Sasaki, Tomoyuki Saito, Taeko Otani, Sachimi Jinbo, Nahoko Ikeda, Masanobu Kawakami, San-e Ishikawa

**Affiliations:** 1 Department of Medicine, Jichi Medical University Saitama Medical Center, 1-847 Amanuma Omiya-ku Saitama, Saitama, Japan

**Keywords:** Osteoprotegerin, Vascular calcification, Atherosclerosis, Type 2 diabetes mellitus

## Abstract

**Background:**

Osteoprotegerin is a member of the tumor necrosis factor-related family and inhibits RANK stimulation of osteoclast formation as a soluble decoy receptor. The goal of this study was to determine the relationship of serum osteoprotegerin with vascular calcification in patients with type 2 diabetes.

**Methods:**

The subjects were 124 patients with type 2 diabetes mellitus, including 88 males and 36 females with a mean (± SD) age of 65.6 ± 8.2 years old. Serum levels of osteoprotegerin, osteocalcin, fibroblast growth factor 23 (FGF23), 25-hydroxyvitamin D3 and adiponectin were measured by ELISA. Vascular calcification in the cervical artery was examined by ultrasound sonography. The subjects were divided into 4 quartiles depending on serum osteoprotegerin levels.

**Results:**

Vascular calcification was significantly higher in the 4th quartile and significantly lower in the 1st quartile of serum osteoprotegerin levels, compared to other quartiles. There were no differences in serum osteoprotegerin and vascular calcification among patients with different stages of diabetic nephropathy, but serum FGF23 levels were elevated in those with stage 4 diabetic nephropathy. Simple regression analysis showed that serum osteoprotegerin levels had significant positive correlations with age, systolic blood pressure and serum adiponectin levels, and significant negative correlations with BMI and serum 25-hydroxyvitamin D3.

**Conclusions:**

These findings suggest that elevated serum osteoprotegerin may be involved in vascular calcification independently of progression of diabetic nephropathy in patients with type 2 diabetes.

## Background

Atherosclerosis, macroangiopathy and microangiopathy are major prognostic factors in diabetes. Vascular endothelial impairment is the initial pathological change and is profoundly involved in development of atherosclerosis [1-4]. Progression of diabetic nephropathy, including microalbuminuria, is a risk factor for atherosclerosis in type 2 diabetes mellitus [5,6] and diabetic patients with advanced nephropathy (particularly those on dialysis with end-stage kidney disease) often have vascular calcification [7-10]. However, the duration of diabetes mellitus is not closely related to the extent of vascular calcification, and this condition may be more strongly related to biochemical changes.

The RANK/RANKL interaction induces osteoclast formation in vascular smooth muscle cells such as those in bone [11]. Osteoprotegerin, a member of the tumor necrosis factor (TNF)-related family [12], binds to RANKL as a soluble decoy receptor, and consequently inhibits RANK stimulation of osteoclast formation [13]. This suggests that interactions among RANK, RANKL and osteoprotegerin influence osteoclastogenesis and vascular calcification. Therefore, in the present study, we examined the relationship of osteoprotegerin with vascular calcification in patients with type 2 diabetes without advanced nephropathy.

## Methods

### Subjects

A total of 124 patients with type 2 diabetes mellitus at the outpatient clinic of Jichi Medical University Saitama Medical Center were enrolled in the study between March 2006 and October 2011. The subjects included 88 males and 36 females, and had a mean (± SD) age of 65.6 ± 8.2 years old (range: 44 to 82 years old). Type 2 diabetes was diagnosed using Japan Diabetes Society criteria. The mean hemoglobin A1c (HbA1c: NGSP) was 7.7 ± 1.4% and the duration of diabetes mellitus was 14.7 ± 8.2 years. Of the 124 subjects, 65 had hypertension, 74 had dyslipidemia, 35 were obese, 40 were current smokers, 46 had diabetic retinopathy, 64 had diabetic nephropathy, and 56 had diabetic neuropathy. Patients with end-stage kidney diseases, use of nitroglycerin as maintenance medication, infectious disease, malignancy, and a history of intrapelvic surgery were excluded from the study.

Blood samples were collected from subjects in the sitting position at a visit to the outpatient clinic after an overnight fast. HbA1c (NGSP), serum total cholesterol, high-density lipoprotein cholesterol, low-density lipoprotein cholesterol, triglyceride, calcium, phosphorus, creatinine, serum osteoprotegerin, osteocalcin, fibroblast growth factor (FGF) 23, 25-hydroxyvitamin D3, and adiponectin were measured in these samples. Urine samples were collected to determine urinary excretion of albumin. Ultrasound sonography of the cervical carotid artery and endothelial function tests of flow-mediated dilatation (FMD) and nitroglycerine-mediated dilatation (NMD) were also performed. The study was approved by the ethical committee of Jichi Medical University for human studies. Informed consent was obtained from all subjects.

Regarding risk factors for atherosclerosis, hypertension was defined as systolic blood pressure > 140 mmHg, diastolic blood pressure > 90 mmHg, or a history of administration of antihypertensive agents; dyslipidemia as a total cholesterol level >2 20 mg/dl, high-density lipoprotein cholesterol level < 40 mg/dl, and triglyceride level > 150 mg/dl, or a history of administration of statins or fibrates; obesity as BMI > 25 (Japanese patients); and a current smoker as a subject who had smoked more than one cigarette per day within the last 3 months.

For progression of nephropathy, the subjects were divided into stages 1–5 based on the classification of diabetic nephropathy by the Research Committee of the Japanese Ministry of Health, Labor, and Welfare for Disorders of Diabetes Mellitus.

### Measurements

Blood samples were collected into tubes and centrifuged at 3,000 rpm at 4°C for 15 min. The supernatants were decanted and frozen at −80°C until assayed. HbA1c was measured by HPLC using an ADAMS™ A1c HA-8160 instrument (Arkray, Kyoto, Japan). The value for HbA1c (%) is estimated as a National Glycohemoglobin Standardization Program (NGSP) equivalent value (%), calculated by the formula HbA1c (%) = HbA1c (Japan Diabetes Society, JDS) (%) + 0.4% [14]. ELISA kits were used to measure the serum levels of osteoprotegerin (Bio Vendor, Modrice, Czech Republic), osteocalcin (Biomedical Technologies Inc., Stoughton, MA, USA), FGF23 (Kainos, Tokyo, Japan), 25-hydroxyvitamin D3 (Immundiagnostik AG., Bensheim, Germany), and adiponectin (Otsuka Pharmaceutical Co., Tokyo, Japan). The minimal levels of detection and interassay and intraassay coefficients of variation were 0.2 pg/ml, 5.8% and 3.5%, respectively, for osteoprotegerin; 0.5 ng/ml, 10.5% and 7.0%, respectively, for osteocalcin; 3 pg/ml, 2.6% and 2.8%, respectively, for FGF23; 12 nmol/l, 7.0% and 7.0%, respectively, for 25-hydroxyvitamin D3; and 0.375 μg/ml, < 10% and < 10%, respectively, for adiponectin. Urinary excretion of albumin was determined by a latex agglutination immunoassay (Eiken, Tokyo). Renal function was calculated as the estimated glomerular filtration rate (eGFR) using the Modification of Diet in Renal Disease equation (MDRD) revised for Japanese subjects by the Japan Society of Nephrology.

### Flow-mediated dilatation (FMD)

Endothelial function was evaluated by FMD, which indicates an arterial response to reactive hyperemia causing endothelial-dependent dilatation [15,16]. FMD of the right brachial artery was determined by one investigator (AA) between 16:00 and 18:00 hours after a 15-minute rest, using a 12-MHz ultrasound instrument (UNEXEF18G, UNEX Corp., Nagoya, Japan), as previously described [17]. The brachial artery was imaged in the longitudinal section just above the antecubital fossa and internal diameters from the anterior to posterior intimal interfaces were measured at end-diastole using B-mode imaging as baseline. A pneumatic cuff was inflated on the forearm at a pressure of more than 50 mmHg higher than systolic blood pressure for 5 min. The intravascular blood flow velocities and vessel diameter were measured from 20 to 120 s after cuff deflation in the same manner as that as baseline. FMD was calculated as the % increase in vessel diameter following reactive hyperemia: (maximum diameter following reactive hyperemia-baseline diameter)/baseline diameter ×100. At 15 min after the FMD measurement, NMD (endothelium-independent dilatation) was measured following administration of sublingual nitroglycerin 0.3 mg-spray. The maximum vessel diameter produced by nitroglycerin was measured 4 min after administration and NMD was calculated.

### Carotid artery sonogram

A carotid artery sonogram for determination of intima-media thickness (IMT) and arterial wall calcification was performed according to the standard method for ultrasound evaluation of carotid artery lesions [18]. Briefly, measurements of IMT in bilateral carotid arteries were obtained in each subject using a 7.5-MHz ultrasound instrument (Prosound SSD-4000SV, Hitachi Aloka Medical, Tokyo, Japan). The IMT was defined as the distance between the leading edge of the lumen-intima echo and the leading edge of the media-adventitia echo. The mean IMT was obtained from measurements performed on the right and left common carotid artery, excluding the bulbus, as an average of 3 points after measurement of IMT at the point of max IMT and two points on both sides (each 1 cm distant from the point of max IMT). Arterial calcification was determined at the same points by evaluating the medial calcification (Monckeberg’s arteriosclerosis) and intimal calcification (atherosclerosis).

### Statistical analysis

Data are expressed as mean ± SEM. Values were analyzed by Student t-test and one-way analysis of variance (ANOVA) to compare differences between the groups. Corresponding data were analyzed by Student paired t-test. Categorical data were analyzed by a chi-square test for independence. For regression analysis, log transformation was used to normalize the distribution of serum levels of osteoprotegerin, FGF23, 25-hydroxyvitamin D3, and adiponectin. Simple linear regression analysis was performed to calculate correlation coefficients. All calculations were performed using SPSS® Statistics 18.0 (Japan IBM., Tokyo, Japan) and a two-tailed p value <0.05 was considered to be statistically significant.

## Results

**Figure 1:**
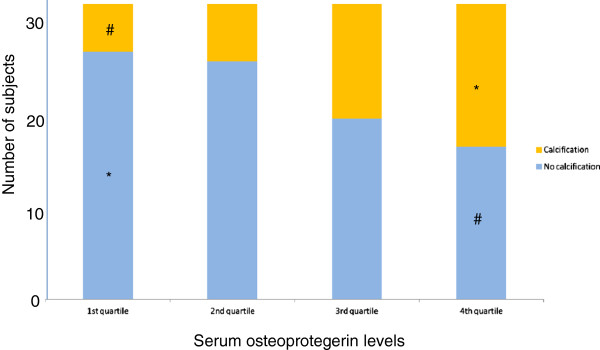
**Presence or absence of cervical arterial calcification in quartiles based on serum osteoprotegerin levels in 124 patients with type 2 diabetes.** P = 0.015 by chi-square test. Serum osteoprotegerin levels in the 1st, 2nd, 3rd and 4th quartiles were <5.6, 5.6-8.4, 8.5-52.7, and 52.8-9027 pg/ml, respectively. * Number of subjects was significantly greater than in other quartiles. # Number of subjects was significantly less than in other quartiles.

The subjects were divided into quartiles using levels of osteoprotegerin, osteocalcin, FGF23, and 25-hydroxyvitamin D3, and the presence or absence of cervical arterial calcification was evaluated in each quartile (Table 1). Based on the serum osteoprotegerin levels, significantly greater vascular calcification was present in subjects in the 4th quartile and significantly less vascular calcification was present in those in the 1st quartile compared to other quartiles (Table 1). The reverse result was found for the absence of calcification (Figure 1). The quartiles based on serum osteocalcin, FGF23 and 25-hydroxyvitamin D3 levels did not show any relationship with vascular calcification.

**Table 1:** Presence or absence of cervical arterial calcification in quartiles based on serum osteoprotegerin, osteocalcin, FGF23, and 25-hydroxyvitamin D3 levels in 124 patients with type 2 diabetes

	**1st quartile**	**2nd quartile**	**3rd quartile**	**4th quartile**
Serum osteoprotegerin (pg/ml) ^a^	<5.6	5.6-8.4	8.5-52.7	52.8- 9027
No calcification, n	26*	25	19	16^#^
Calcification, n	5#	6	12	15*
Serum osteocalcin (ng/ml) ^b^	0.77-0.82	0.83-0.92	0.93-1.53	1.60-10.56
No calcification, n	24	18	21	23
Calcification, n	7	13	10	8
Serum FGF23 (pg/ml) ^c^	10.0-22.7	22.9-29.0	29.7-40.7	42.0- 148.0
No calcification, n	24	20	19	23
Calcification, n	7	11	12	8
Serum 25-hydroxyvitamin D3 (nmol/l) ^d^	29.2-48.1	48.6-77.3	78.6-119.1	121.1-239.3
No calcification, n	20	19	25	22
Calcification, n	11	12	6	9

^a^ P=0.015 by chi-square test. *: Number of subjects significantly greater than other quartiles. ^#^: Number of subjects significantly less than other quartiles.

^b^ P=0.278 by chi-square test.

^c^ P=0.499 by chi-square test.

^d^ P=0.364 by chi-square test.

Clinical and laboratory data for the quartiles based on serum osteoprotegerin levels are shown in Table 2. Age and serum adiponectin levels increased with higher serum osteoprotegerin levels (P = 0.001 and P = 0.002, respectively, in trend tests), and serum 25-hydroxyvitamin D3 levels were significantly lower in the 4th quartile (P =0.002 ).

**Table 2:** Clinical and laboratory data for 124 patients with type 2 diabetes divided into quartiles of serum osteoprotegerin levels (< 5.6; 5.6-8.4; 8.5-52.7; and 52.8-9027 pg/ml)

	**1st quartile**	**2nd quartile**	**3rd quartile**	**4th quartile**	**P value**
Subjects (male/female)	31(25/6)	31(20/11)	31(23/8)	31(20/11)	
Age (years)	61.0±1.2	66.3±1.4*	65.9±1.5	69.6±1.4**	0.001
Height (cm)	165.3±1.4	158.3±1.4**	164.6±1.1^##^	158.8±1.5**^,$^	0.001
Weight (kg)	68.6±2.4	59.6±1.7*	66.7±2.5	60.0±1.7*	0.003
BMI	24.9±0.7	23.7±0.5	24.5±0.7	23.6±0.5	0.366
Duration of diabetes mellitus (years)	12.0±1.4	16.9±1.6	14.7±1.4	15.2±1.4	0.142
Systolic blood pressure (mmHg)	127.0±2.6	133.6±2.5	136.1±4.6	140.7±2.6*	0.029
Diastolic blood pressure (mmHg)	74.7±1.5	75.0±1.6	74.9±1.7	75.7±1.7	0.975
FMD (%)	4.0±0.5	4.4±0.4	4.2±0.4	3.0±0.7	0.178
Mean IMT (mm)	0.95±0.05	1.07±0.06	1.13±0.07	1.04±0.09	0.337
HbA1c (NGSP) (%)	8.0±0.4	7.6±0.2	7.9±0.2	7.4±0.2	0.371
Total cholesterol (mg/dl)	192.1±7.7	191.6±4.6	188.1±6.4	192.4±4.7	0.953
Triglyceride (mg/dl)	136.6±12.9	129.7±12.6	120.5±9.0	137.1±10.8	0.709
HDL-Cholesterol (mg/dl)	47.8±2.3	51.4±2.7	55.2±2.3	51.7±2.3	0.210
LDL-Cholesterol (mg/dl)	111.7±7.4	113.8±4.3	109.0±5.2	113.0±5.2	0.937
Creatinine (mg/dl)	0.78±0.03	0.79±0.03	0.81±0.04	0.83±0.05	0.721
eGFR (ml/min/1.73m^2^)	79.5±3.7	70.0±3.0	74.4±4.1	69.2±3.4	0.159
Albuminuria (mg/g creatinine)	75.4±27.9	146.8±37.6	173.7±43.6	117.4±53.6	0.357
Adiponectin (μg/ml)	5.8±0.7	8.5±1.1	9.0±1.6	12.2±1.0**	0.002
Osteocalcin (ng/ml)	1.6±0.2	1.7±0.4	1.4±0.2	1.9±0.3	0.596
25-hydroxyvitamin D3 (nmol/l)	93.1±7.7	103.4±7.3	93.9±10.1	61.9±5.6*^,##,$^	0.002
FGF23 (pg/ml)	32.7±2.8	31.3±3.1	39.0±4.5	34.8±2.2	0.370
Ca (mg/dl)	9.3±0.05	9.4±0.06	9.4±0.07	9.3±0.07	0.499
iP (mg/dl)	3.6±0.09	3.6±0.09	3.6±0.08	3.5±0.08	0.937

Values are shown as mean±SE and were analyzed by ANOVA. eGFR: estimated glomerular filtration rate. * P< 0.05 vs. 1st quartile. ** P < 0.01 vs. 1st quartile. # P < 0.05 vs. 2nd quartile. ## P < 0.01 vs. 2nd quartile. $ P < 0.05 vs. 3rd quartile. $$ P<0.01 vs. 3rd quartile.

Vascular calcification and other parameters in the subjects based on clinical stages of diabetic nephropathy, a microvascular angiopathy, are shown in Table 3. Serum creatinine and albuminuria increased and eGFR decreased with progression of nephropathy. Serum FGF23 was elevated in subjects with stage 4 nephropathy (P = 0.007), whereas osteoprotegerin, osteocalcin, and 25-hydroxyvitamin D3 did not differ significantly among the stages of diabetic nephropathy. There was also no tendency for alterations in vascular calcification among the stages of nephropathy.

**Table 3:** Laboratory data for 124 patients with type 2 diabetes according to progression of diabetic nephropathy

	**Stage 1**	**Stage 2**	**Stage 3**	**Stage 4**	**P value**
Subjects (male/female)	59(42/17)	40(24/16)	19(17/2)	6(5/1)	
Serum creatinine (mg/dl)	0.73±0.02	0.81±0.03	0.87±0.03*	1.2±0.10**^,##,$$^	< 0.001
eGFR (ml/min)	80.1±2.6	69.3±2.9*	68.7±4.0	46.5±3.2**^,#^	<0.001
Albuminuria (mg/g creatinine)	12.4±1.1	95.0±10.7**	483.8±63.6**^,##^	538.0±98.4**^,##^	<0.001
Serum osteoprotegerin (pg/ml)	183.8±117.5	329.6±227.4	168.1±87.2	59.8±27.6	0.876
Serum osteocalcin (ng/ml)	1.58±0.21	1.79±0.26	1.35 ±0.32	2.06 ±0.76	0.696
Serum 25-hydroxyvitamin D3 (nmol/l)	85.3±5.71	86.2±6.52	103.3±14.9	81.1±18.8	0.516
Serum FGF23 (pg/ml)	32.6±1.83	33.2±3.53	36.1±3.57	58.7±7.08**	0.007
No calcification, n	41	26	13	5	
Calcification, n	18	14	4	1	

Values are shown as mean ± SE and were analyzed by ANOVA. * P < 0.05 vs. 1st quartile. ** P < 0.01 vs. 1st quartile. # P < 0.05 vs. 2nd quartile. ## P < 0.01 vs. 2nd quartile. $ P < 0.05 vs. 3rd quartile. $$ P < 0.01 vs. 3rd quartile.

**Figure 2:**
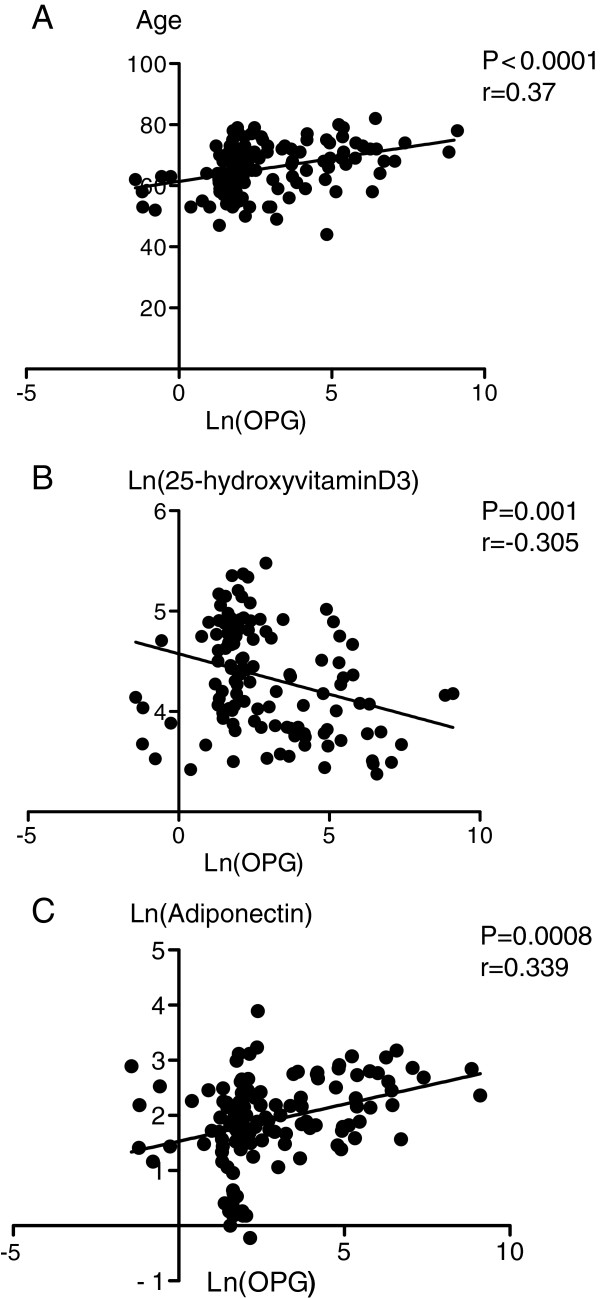
Relationships of serum osteoprotegerin (OPG) levels with age (A), serum 25-hydroxyvitamin D3 (B), and serum adiponectin (C) in the patients with type 2 diabetes.

The results of simple regression analysis of variables associated with serum osteoprotegerin levels in the subjects are shown in Table 4 and Figure 2. Serum osteoprotegerin levels were positively correlated with age (p < 0.0001), systolic blood pressure (P=0.03), and serum adiponectin levels (P = 0.0008); and negatively correlated with BMI (P = 0.045) and serum 25-hydroxyvitamin D3 (P = 0.001).

**Table 4:** Simple linear regression analysis of variables with possible associations with serum osteoprotegerin levels in patients with type 2 diabetes

	**P value**	**r**
Age	<0.0001**	0.367
BMI	0.045*	−0.181
Systolic blood pressure	0.03*	0.195
Diastolic blood pressure	0.722	−0.032
Duration of diabetes mellitus (years)	0.255	0.104
HbA1c (NGSP)	0.072	−0.163
Total-Cholesterol	0.972	−0.003
Triglyceride	0.927	0.008
HDL-Cholesterol	0.310	0.093
LDL-Cholesterol	0.809	−0.022
Creatinine	0.878	0.014
Ln (FGF23)	0.373	0.081
Ln (25-hydroxyvitamin D3)	0.001**	−0.305
Osteocalcin	0.422	0.073
Ln (FMD)	0.294	−0.096
Mean IMT	0.976	0.002
Ln (Adiponectin)	0.0008**	0.339
EPC	0.330	−0.134
Ln Ca	0.217	−0.112
iP	0.983	−0.002
Ualb	0.634	0.049
CVRR	0.362	0.087

## Discussion

The results of the study demonstrate that vascular calcification is closely associated with serum osteoprotegerin levels in patients with diabetes [19], but is not linked to other bone-related humoral factors including osteocalcin, FGF23, and 25-hydroxyvitamin D3. Osteoprotegerin may directly affect osteoblastic changes of vascular smooth muscle cells and is not mediated through other factors or ions [12,13]. This differs from the effect of FGF23 on vascular calcification, since FGF23 regulates phosphate metabolism in kidney and promotes vascular calcification in association with phosphate [20]. In this study, we examined diabetic patients with mild or moderate renal impairment, including subjects with diabetic nephropathy of stages 1–4, since patients with advanced nephropathy may have vascular calcification without any interaction with cytokines [7].

FMD is a good indicator for vascular endothelial function and was found to be unrelated to serum osteoprotegerin or vascular calcification. These findings indicate that elevated serum osteoprotegerin may be involved in vascular calcification in patients with diabetes, independently of progression of diabetic nephropathy. However, because the study was performed as a cross-sectional observation, the finding of an association of serum osteoprotegerin and vascular calcification is limited and cannot suggest causality. However, osteoprotegerin may be clinically useful as a biochemical marker of vascular damage and overall burden of atherosclerotic disorders. In fact, serum osteoprotegerin is known to be associated with carotid and peripheral arterial disease in patients with type 2 diabetes [21] and is inversely associated with aortic distensibility [22]. Osteoprotegerin is also an independent predictor of coronary artery disease in asymptomatic type 2 diabetic patients with microalbuminuria [23] and is also predictive of the long-term outcome in patients with ST-elevation myocardial infarction treated with percutaneous coronary intervention [24].

Osteoprotegerin is a member of the TNF-related family [12] and exerts its major biological action through binding to RANKL as a soluble decoy receptor, with resulting inhibition of RANK stimulation of osteoclast differentiation and bone resorption [13,25]. RANKL and osteoprotegerin are expressed in osteoblasts, and the receptor RANK is expressed in osteoclasts cells [25]. In vascular beds, endothelial cells and vascular smooth muscle cells produce osteoprotegerin, but do not produce RANK and RANKL [11,13,26,27]. However, both RANKL and RANK expression have been detected in atherosclerotic lesions [28-30]. This may indicate that the RANK-RANKL interaction induces osteoclast formation and that osteoprotegerin blocks this interaction to reduce arterial calcification [11,31,32]. Therefore, vascular calcification linked to RANK-RANKL is independent of advanced vascular damage related to a long duration of diabetes, progression of diabetic microvascular complications, and abnormal metabolism of calcium or phosphate associated with FGF23, 25-hydroxyvitamin D3 and other factors. In the present study, we could not determine how the RANK/RANKL system participates in development of osteoclast differentiation in the vasculature in the non-advanced stage of diabetes mellitus. However, our findings suggest that vascular calcification may develop in patients with diabetes with high serum osteoprotegerin levels, even if microvascular and macrovascular disorders do not become manifest. The elevation of serum osteoprotegerin might be associated with underlying alterations in RANK/RANKL interactions in the vascular wall.

Vascular calcification is common in patients with diabetes, and especially in those with diabetic nephropathy [8-10]. We have shown that coronary artery calcification is significantly increased in advanced diabetic nephropathy based on histology of intravascular ultrasound [7]. In such cases, abnormal calcium or phosphorus metabolism may also be involved in vascular calcification, in association with FGF23 or 25-hydroxyvitamin D3 [20,33]. As shown in Table 3, we found no relationship of vascular calcification or serum osteoprotegerin levels with diabetic nephropathy, and only 6 of the 124 subjects in the study had stage 4 diabetic nephropathy. Thus, most had no or mild nephropathy, making it evident that progression of vascular calcification is not simply associated with diabetic nephropathy.

BMI, systolic blood pressure, adiponectin and 25-hydroxyvitamin D3 were among the factors correlated with serum osteoprotegerin in simple linear regression analysis. There was a highly significant positive correlation between serum adiponectin and serum osteoprotegerin. In bone tissue, adiponectin stimulates RANKL in osteoclasts, thus inducing osteoclastogenesis [34]. In contrast, adiponectin inhibits osteoprotegerin in osteoblasts [34]. The positive correlation between serum adiponectin and osteoprotegerin [35] indicates that serum adiponectin may interact with osteoprotegerin for modulating osteoclast formation. Serum osteoprotegerin had a negative correlation with serum 25-hydroxyvitamin D3. Several studies have examined the effect of vitamin D3 on osteoprotegerin synthesis [36] and it has been shown that active 1α, 25-hydroxyvitamin D3 upregulates the level of RANKL, but downregulates osteoprotegerin expression in human periodontal ligament cells by accelerating degradation of osteoprotegerin mRNA and transrepressing the osteoprotegerin gene [37,38]. This indicates that active vitamin D3 promotes osteoclastogenesis. Similar interactions of osteoprotegerin with adiponectin and 25-hydroxyvitamin D3 may occur in vascular tissues, particularly with regard to the vascular calcification evaluated in this study.

## Conclusions

Serum osteoprotegerin levels were closely associated with vascular calcification in patients with type 2 diabetes. Serum osteoprotegerin interacts with RANK/RANKL as a soluble decoy receptor to prevent osteoclast differentiation, and the elevation of serum osteoprotegerin might be linked to underlying alterations in RANK/RANKL interactions in the vascular wall. The levels of vascular calcification and serum osteoprotegerin were independent of progression of diabetic nephropathy. These findings indicate that elevated serum osteoprotegerin may be involved in vascular calcification in patients with type 2 diabetes mellitus.

## Abbreviations

FGF 23: Fibroblast growth factor 23; OPG: Osteoprotegerin; TNF: Tumor necrosis factor; FMD: Flow-mediated dilatation; NMD: Nitroglycerine-mediated dilatation; IMT: Intima-media thickness.

## Competing interests

The authors declare that they have no competing interests.

## Authors’ contributions

AA, MM and SI designed the study. AA, MM, TO, SJ and NI collected the data. AA, MM, TA, AI, MS, TS, MK and SI analyzed the data. AA, MM and SI wrote the first draft of the manuscript. All authors reviewed and edited the manuscript, and approved the final version of the manuscript for publication.
